# Delivering Therapy to the Olfactory Cleft: A Comparison of the Various Methods of Administering Topical Nasal Medications

**DOI:** 10.7759/cureus.53523

**Published:** 2024-02-03

**Authors:** Patricia T Jacobson, Lucas G Axiotakis, Brandon J Vilarello, David A Gudis, Daniel B Spielman, Nathan Yang, Carol H Yan, Zach M Soler, Joshua M Levy, Nicholas R Rowan, Alexandria L Irace, Jonathan B Overdevest

**Affiliations:** 1 Department of Otolaryngology-Head and Neck Surgery, NewYork-Presbyterian/Columbia University Irving Medical Center, New York, USA; 2 Otolaryngology, Vagelos College of Physicians and Surgeons, Columbia University, New York, USA; 3 Department of Otolaryngology-Head and Neck Surgery, Emory University School of Medicine, Atlanta, USA; 4 Department of Otolaryngology-Head and Neck Surgery, The University of California, San Diego School of Medicine, La Jolla, USA; 5 Department of Otolaryngology-Head and Neck Surgery, Medical University of South Carolina, Mt. Pleasant, USA; 6 Department of Otolaryngology-Head and Neck Surgery, Johns Hopkins University School of Medicine, Baltimore, USA

**Keywords:** chronic rhinosinusitis, exhalation delivery system, kaiteki, nasal spray, olfactory cleft

## Abstract

Background and objective

Chronic rhinosinusitis (CRS) is an inflammatory condition affecting the nasal mucosa, and it causes olfactory dysfunction (OD) in up to 78.2% of patients. Corticosteroids are the mainstay of treatment to shrink nasal polyposis, reduce inflammation, and improve olfactory function. While many delivery methods for topical nasal corticosteroids exist, there is scarce data on the efficacy of the various medication delivery methods to the olfactory cleft (OC). In light of this, this study aimed to compare the following delivery methods to the OC: conventional nasal spray (NS), nasal drops in the Kaiteki position (KP), and exhalation delivery system (EDS).

Methods

We evaluated 16 sinonasal cavities from eight cadaver specimens in this study. Each sinonasal cavity was administered fluorescein dye solution via NS, KP, and EDS. Following administration, nasal endoscopy was employed to capture staining patterns in the OC. OC staining was rated with scores ranging from 0 (no staining) to 3 (heavy staining) after each administration of dye solution. Mean OC staining ratings were calculated and compared using the Kruskal-Wallis rank sum test and the Wilcoxon signed-rank test.

Results

The mean OC staining score for the different delivery methods was as follows - NS: 1.095 ± 1.008, EDS: 0.670 ± 0.674, and KP: 2.038 ± 1.097. Nasal drops in the KP had a significantly higher staining score compared to NS (p=0.041) and EDS (p=0.003). However, there was no significant difference in staining scores between NS and EDS.

Conclusions

Nasal drops in the KP are more effective at reaching the OC than NS or EDS and should be considered as a first-line modality for administering topical medications when treating OD.

## Introduction

Chronic rhinosinusitis (CRS) is an inflammatory condition affecting the nasal mucosa that can occur with nasal polyposis (CRSwNP) and without (CRSsNP) [1]. Both types of CRS can cause various rhinologic symptoms including rhinorrhea, congestion, facial pain, and olfactory dysfunction (OD) [1]. Previous research has found that up to 78.2% of individuals with CRS suffer from OD [2]. Due to the symptom burden in people with CRS, the condition is associated with a diminished quality of life, increased risk for depression, and poor sleep quality [3]. Hence, timely and proper treatment of CRS is extremely important to control the associated symptoms and alleviate their burden on individuals’ daily activities and quality of life.

Corticosteroids are a mainstay of treatment for CRSwNP and CRSsNP [4] and act to decrease the inflammation that accompanies the disease. While steroids can be administered orally and intranasally, they are preferred to be administered intranasally as this helps prevent the systemic side effects that often follow steroid use [5]. The goal of intranasal steroid use is to reduce nasal polyposis and inflammation to improve olfactory function. However, studies have shown that intranasal steroids lack the potency of systemic steroids [6,7]. This disparity may be exacerbated by the poor penetration of the medication into the appropriate areas within the nasal cavity [7,8]. For intranasal medications to effectively improve olfactory function in diseases such as CRS, medications must reach regions responsible for facilitating odorant airflow and be deposited on the olfactory epithelium, namely the internal nasal valve, middle meatus, and olfactory cleft (OC) [8,9]. The effective delivery of medication to OC for improving olfactory function is especially important since OD secondary to CRS can impact patients' appetite, social relationships, and safety [10].

Various methods for intranasal medication delivery have been developed, including nasal sprays (NS), nasal drops, sinus rinses, and more recently, exhalation delivery systems (EDS). In all these administration methods, especially nasal drops, head positioning impacts delivery [8,11,12]. According to research investigating the best head positions for nasal-drop access to the OC, the Kaiteki position (KP) is more effective and comfortable compared to alternatives such as the head-leaning-forward position [12,13], thereby improving patient compliance [11,14]. EDS uses a timed exhalation method to bypass some of these head-positioning challenges and utilizes positive pressure to broadly disperse medications in the nasal cavity, especially to the superior sinonasal regions [15].

There is sparse research directly comparing these commonly used methods of intranasal steroid administration for the delivery of medication to the OC. Our study aims to fill this void by analyzing the variations in patterns of dye solution deposition in the OC following delivery using NS, KP, and EDS. We believe our findings will help guide clinicians on how to best target the OC topically when treating smell disorders.

## Materials and methods

**Figure 1:**
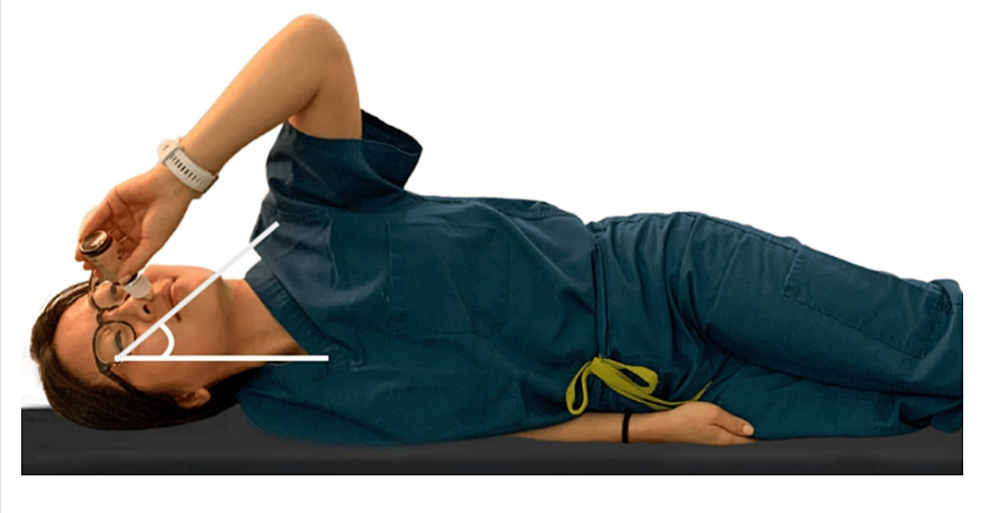
The Kaiteki position for the administration of nasal drops This method involves the patient lying on their side with the chin turned 20°-40° upwards and the head tilted 20°-30° downwards

This study involved 16 sinonasal cavities from eight cadaver specimens, without any significant anatomic abnormalities or sinonasal disease. The age of the cadaver specimens was not provided. Fluorescein dye was diluted to produce a solution concentration of 0.1 mg/mL. The diluted solution was used to fill the NS, EDS, and nasal drop vials for administration. With the cadaver head placed in a neutral position, NS was administered according to the manufacturer's instructions. Two sprays of the fluorescein dye solution were applied in each nostril bilaterally, amounting to 0.137 mL of solution delivered to each nostril. EDS administration employed a modified device to enable third-party administration and was administered according to the manufacturer's instructions. The device was placed into the cadaver's nostril with one examiner exhaling through a tube attached to the exhalation mouthpiece to deploy 0.133 mL of dye solution into the nostril, while the other examiner provided a firm seal with occlusion of the contralateral nostril. The same maneuvers were used to administer solution via EDS to the contralateral sinonasal cavity. For nasal drop administration, cadaver heads were placed in the Kaiteki position (Figure 1) and three drops of fluorescein solution, equating to approximately 0.15 mL of solution, were administered from a Pred Forte ophthalmic bottle into each nostril bilaterally.

**Figure 2:**
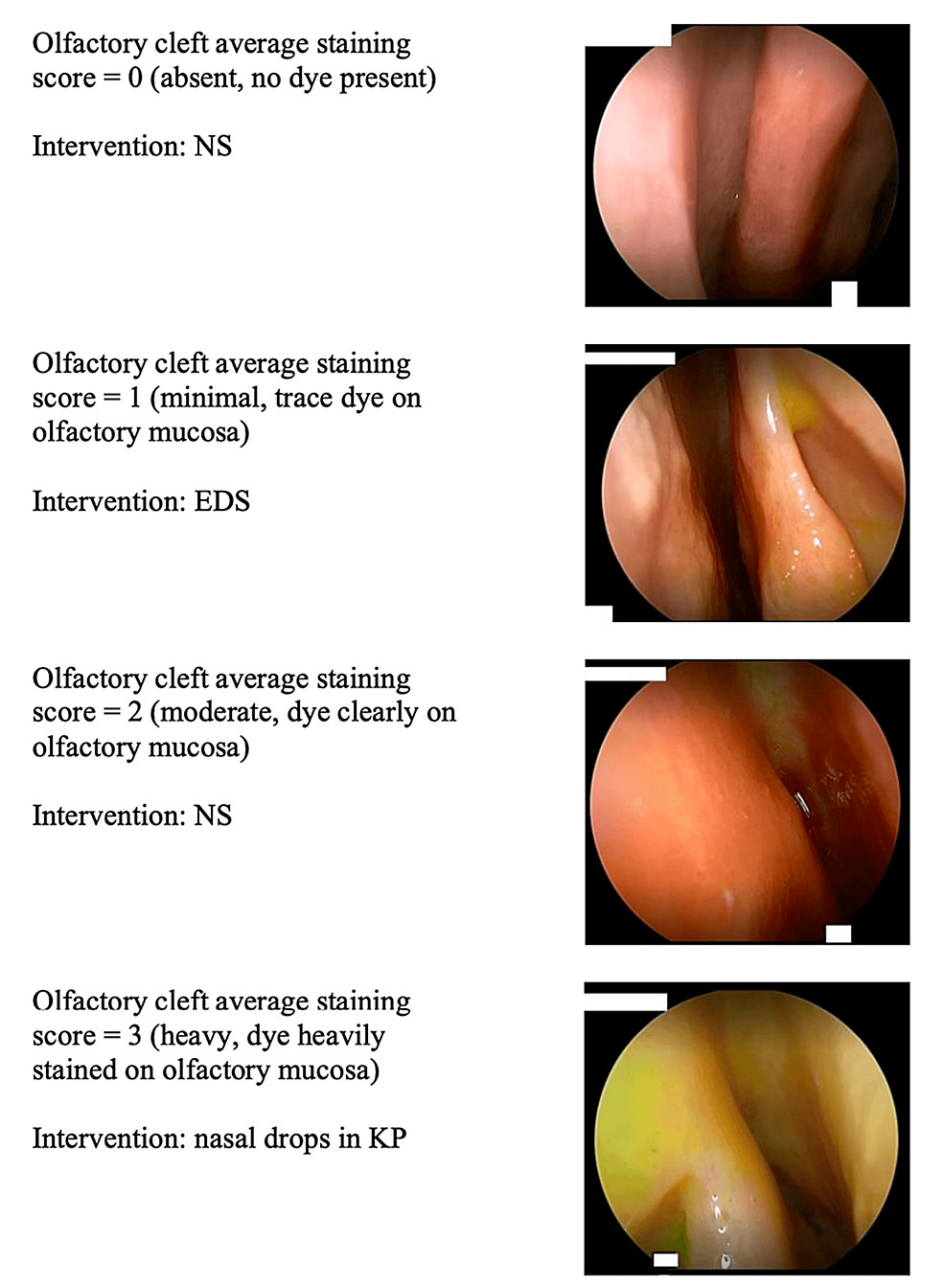
Examples of OC staining EDS: exhalation delivery system; KP: Kaiteki position; NS: nasal spray; OC: olfactory cleft

After each administration of the fluorescein solution, a nasal endoscopy video was used to capture the region of the OC. Each sinonasal cavity was thoroughly irrigated to remove any dye before the next administration of solution and nasal endoscopy was performed after irrigation to verify that there was no remaining stain before the next administration of dye. Representative still images from each video were reviewed by seven trained rhinologists to rate the degree of staining in the OC on a scale of 0-3, where a score of 0 correlated with no visible staining and a score of 3 represented heavy staining of the OC (Figure 2) [16]. Ratings were submitted by each rater through an online Qualtrics platform interface.

This study protocol was reviewed by the Columbia University Irving Medical Center IRB and deemed exempt from requiring ethics approval.

Statistical analysis

The R statistical software was used for statistical analysis. Interobserver reliability was calculated using Cronbach’s alpha statistic. Kruskal-Wallis rank sum test followed by post hoc paired comparisons using the Wilcoxon signed rank test was used for comparisons of means between groups since the data was non-parametric. A p-value <0.05 was considered statistically significant.

## Results

Eight cadaver specimens, comprising both right and left sinonasal cavities (n=16) were exposed to NS, KP, and EDS delivery processes, and data were documented for 15 sinonasal cavities for examination after NS. The Cronbach alpha score for interobserver reliability was 0.97 based on all ratings provided for OC staining observed after NS, EDS, and KP for each cadaver side.

Nasal spray vs. exhalation delivery system vs. nasal drops in the Kaiteki position

**Figure 3:**
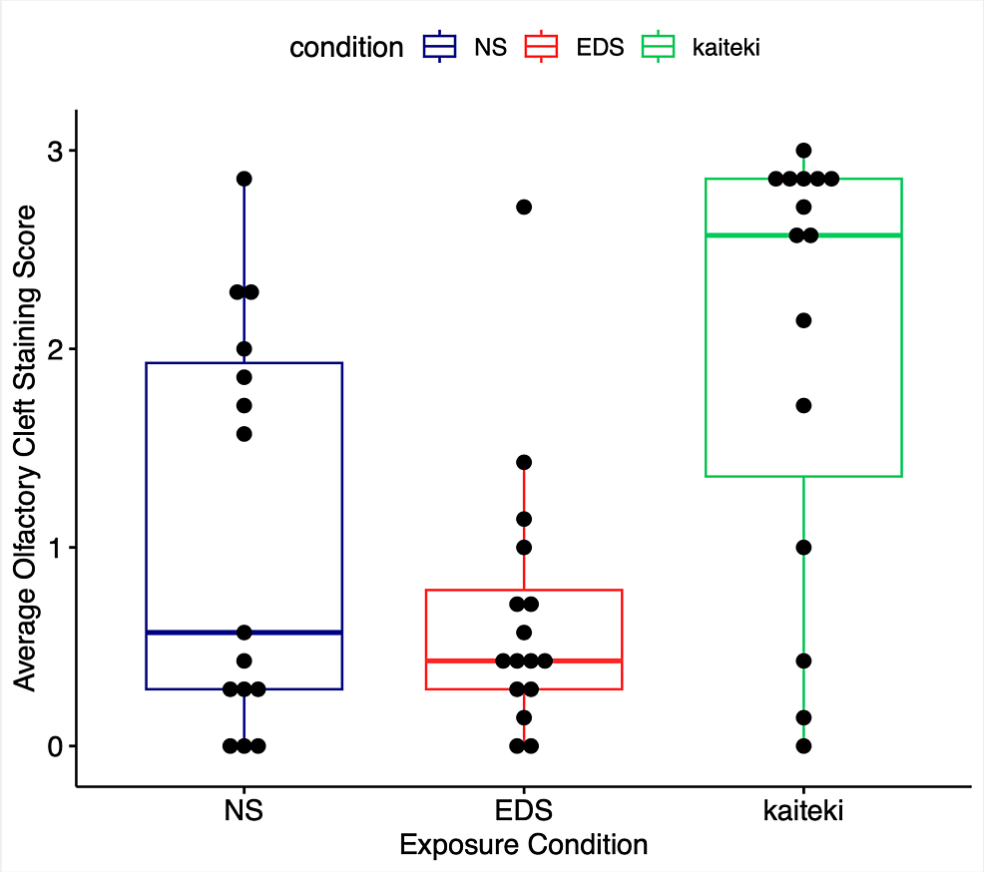
Box plot showing average OC staining score for NS (n=15), EDS (n=16), and KP (n=16) Each dot represents the average rating for a single cadaver EDS: exhalation delivery system; KP: Kaiteki position; NS: nasal spray; OC: olfactory cleft

The mean ratings for OC staining in cadavers after NS, EDS, and KP are shown in Table 1. The mean ratings for each individual sinonasal cavity after NS, EDS, and KP administration as well as the overall mean ratings for each delivery method are shown in Figure 3. KP delivery resulted in a significantly higher mean staining score of the OC based on a paired comparison to NS (p=0.041) and EDS (p=0.003). There was no significant difference in mean staining scores between NS and EDS (p=0.221).

**Table 1:** Average ratings for OC staining among the three different delivery methods OC: olfactory cleft; SD: standard deviation

Delivery method	OC staining score, mean ± SD
Nasal spray (n=15)	1.095 ± 1.008
Exhalation delivery system (n=16)	0.670 ± 0.674
Nasal drops in Kaiteki position (n=16)	2.038 ± 1.097

## Discussion

The results of our study demonstrate that nasal drops administered in KP are significantly more effective at reaching the OC when compared to NS or EDS. KP showed an average score of 2.038 out of 3, demonstrating that there was a large amount of solution reaching the OC, thereby leading to moderate to heavy staining of the OC. These results indicate that in practice, a clinician should consider KP as a first-line modality for the delivery of medications to the OC compared to NS or EDS. These findings also suggest that despite the perceived advantages of nasal sprays in delivering a constant dose of medication evenly to the surface area of the nasal cavity [17] and their easy-to-use application [9], optimal delivery of medication to the OC is provided by KP. This is the first study to engage in a direct comparison of these application methods.

Studies by Mori et al. [11] and Milk et al. [13] have also demonstrated that KP is an efficacious delivery method of medication to the OC. However, these studies did not compare nasal drops with other methods of intranasal medication administration. Another study assessing the delivery of medication to the OC in patients who underwent functional endoscopic sinus surgery found that the use of nasal drops in the vertex-to-floor position delivered a significantly greater amount of solution to the OC compared to NS [18]. Though this head position is different from the one used in our study, both the Kaiteki and vertex-to-floor head positions have been shown to adequately deliver medication to the OC [8,11-13]. Results from these studies further align with our findings, demonstrating the superiority of nasal drops applied via appropriate head positions for delivering medication to the OC. In contrast, a study by Scheibe et al. [19] showed that neither nasal drops nor NS administered in the head-back position were able to reach the OC consistently. These results are more reflective of the shortcomings of this head position, head-back, which has been shown to mainly distribute medication to the nasal floor, further validating the argument that head position is an important factor to be considered when administering nasal drops since they are gravity-dependent [12].

Notably, other studies have demonstrated the clinical efficacy of corticosteroids in olfaction modulation, where patients with CRS who use EDS with fluticasone [15,20] experienced subjective improvement in their sense of smell. Improvement in olfaction in these scenarios may represent a regional decrease in inflammation and improved patency of the internal nasal valve and middle meatal regions with enhanced corticosteroid distribution. Thus, while penetration of the OC with optimal delivery methods may offer the best course for direct treatment of the olfactory epithelium, variations in etiologies of OD and contributions of both conductive and inflammatory effects in rhinologic causes of olfactory loss create further complexities in directing topical care.

Overall, our study's results and prior research support KP as a more effective method for medication delivery to the OC, which may have broader implications when targeting the olfactory epithelium as novel medications become available. The pathophysiology of OD in patients with CRS is not completely understood. While it has been hypothesized that OD results from airflow obstruction often secondary to polyps [21,22], studies have shown residual OD in some individuals even with medical and surgical treatments to reduce or remove polyps [22,23]. It has also been hypothesized that OC inflammation may impair signaling between olfactory receptors and the olfactory cortex [21,22]. As future studies investigate and provide a better understanding of the pathophysiology of OD in CRS, novel treatments that need to be delivered to the OC to improve olfactory function will likely be developed. Therefore, obtaining data that supports KP as the most effective method for delivering medication to the OC is important when considering administration techniques for these future medications.

Our study has a few limitations, including the use of cadavers, which may have resulted in non-physiologic anatomical variations. Additionally, we utilized a limited data set involving only 16 sinonasal cavity sides; however, a priori power analysis assumptions and an ability to discern variations in mode of delivery suggest this sample size to be sufficient despite it possibly limiting the generalizability of our findings. Further studies involving larger sample sizes that compare NS, KP, and EDS methods of intranasal medication delivery should be conducted to validate our findings and better understand whether OD therapy improves with the use of these techniques.

## Conclusions

Based on our findings, nasal drops via KP are a more effective method of intranasal topical solution delivery to the OC than NS or EDS. Hence, KP should be considered as a first-line method of delivery for topical nasal medications when treating sinonasal conditions causing OD.
